# *catena*-Poly[[(3-methyl­sulfanyl-1,2,4-thia­diazole-5-thiol­ato)sodium]di-μ-aqua-κ^4^ *O*:*O*]

**DOI:** 10.1107/S1600536809051721

**Published:** 2009-12-04

**Authors:** Jun-Hong Zhang, Chun-Lin Ma, Ru-Fen Zhang, Hai-Zeng Wang, Guo-Jia Fu

**Affiliations:** a College of Chemistry and Chemical Engineering, Ocean University of China, Qingdao 266100, Shandong, People’s Republic of China; b College of Chemistry and Chemical Engineering, Liaocheng University, Shandong 252059, People’s Republic of China

## Abstract

The crystal structure of the title compound, [Na(C_3_H_3_N_2_S_3_)(H_2_O)_2_]_*n*_, features polymeric chains made up of O⋯O edge-shared NaSN(H_2_O)_4_ units running along the *b* axis. The Na^+^ ion and all non-H atoms of the thia­diazole ligand lie on a mirror plane.

## Related literature

For related structures, see: Guo (2004 ▶); Wang *et al.* (2007 ▶).
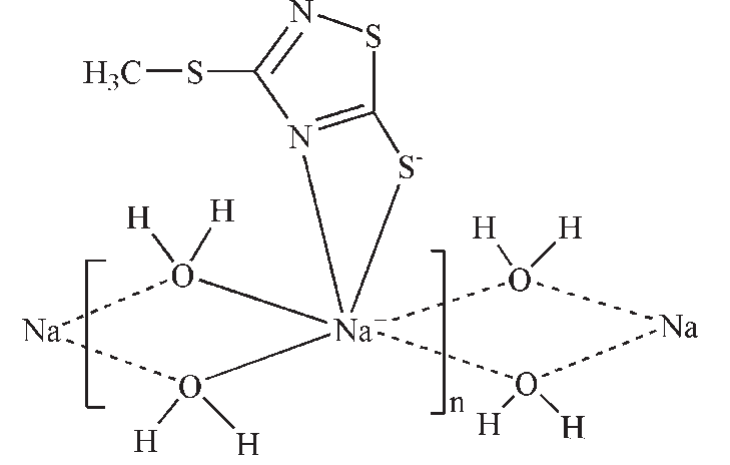


## Experimental

### 

#### Crystal data

[Na(C_3_H_3_N_2_S_3_)(H_2_O)_2_]
*M* *_r_* = 222.28
Monoclinic, 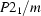

*a* = 7.5794 (8) Å
*b* = 6.9736 (6) Å
*c* = 8.6879 (12) Å
β = 102.027 (1)°
*V* = 449.13 (9) Å^3^
*Z* = 2
Mo *K*α radiation
μ = 0.83 mm^−1^
*T* = 298 K
0.39 × 0.27 × 0.15 mm

#### Data collection

Siemens SMART CCD area-detector diffractometer
Absorption correction: multi-scan (*SADABS*; Sheldrick, 1996 ▶) *T*_min_ = 0.739, *T*_max_ = 0.886
2256 measured reflections
862 independent reflections
728 reflections with *I* > 2σ(*I*)
*R*_int_ = 0.017

#### Refinement

*R*[*F* ^2^ > 2σ(*F* ^2^)] = 0.024
*wR*(*F* ^2^) = 0.064
*S* = 1.07
862 reflections
64 parameters
H-atom parameters constrained
Δρ_max_ = 0.28 e Å^−3^
Δρ_min_ = −0.21 e Å^−3^

### 

Data collection: *SMART* (Siemens, 1996 ▶); cell refinement: *SAINT* (Siemens, 1996 ▶); data reduction: *SAINT*; program(s) used to solve structure: *SHELXS97* (Sheldrick, 2008 ▶); program(s) used to refine structure: *SHELXL97* (Sheldrick, 2008 ▶); molecular graphics: *SHELXTL* (Sheldrick, 2008 ▶); software used to prepare material for publication: *SHELXTL*.

## Supplementary Material

Crystal structure: contains datablocks I, global. DOI: 10.1107/S1600536809051721/ci2968sup1.cif

Structure factors: contains datablocks I. DOI: 10.1107/S1600536809051721/ci2968Isup2.hkl

Additional supplementary materials:  crystallographic information; 3D view; checkCIF report

## Figures and Tables

**Table 1:** Selected bond lengths (Å)

Na1—O1	2.4493 (16)
Na1—N2	2.467 (2)
Na1—O1^i^	2.4736 (16)
Na1—S3	3.1271 (14)

Symmetry code: (i) 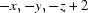.

**Fig. 1.**
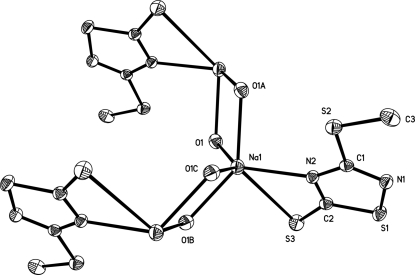
The coordination environment around the Na+ ion. Displacement ellipsoids are drawn at the 30% probability level. [Symmetry codes: (A) -x, -y, 2-z; (B) x, 1/2-y, z; (C) -x, 1/2+y, 2-z].

**Fig. 2.**
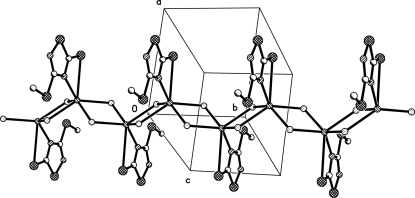
Part of the polymeric chain parallel to the b axis. H atoms have been omitted for clarity.

## supplementary crystallographic information

### Comment

In the title compound (Fig.1), each Na^+^ ion has a six-coordinate environment formed by a S atom and a N atom of the 3-methylsulfanyl-1,2,4-thiadiazole-5-thiolate ligand, and four bridging water O atoms O1, O1A, O1B and O1C. The adjacent NaSNO_4_ units share O1···O1A and O1B···O1C edges, producing chains running along the *b* axis (Fig. 2). Similar chains were found in the crystal structure of sodium carboxynitrobenzoate tetrahydrate (Guo, 2004). The Na—O [2.4493 (16) and 2.4736 (16) Å], Na—S [3.1271 (14) Å] and Na—N [2.467 (2) Å] distances are comparable to those observed in a related structure (Wang *et al.*, 2007).

### Experimental

To a solution of 3-methylmercapto-5-mercapto-1,2,4-thiadiazole (10 mmol) in 60 ml of doubly-distilled water, a solution of an equimolar amount (10 mmol) of sodium hydroxide in 40 ml of doubly-distilled water was added dropwise at room temperature. After vigorous stirring for 6 h, the resulting mixture was evaporated *in vacuo* to a volume of about 20 ml and filtered hot. The filtrate was then set aside for crystallization at room temperature. Two weeks later, colourless single crystals suitable for X-ray diffraction were obtained.

### Refinement

H atoms of the water molecules were initially located a difference Fourier map and later refined using a riding model with O-H = 0.85Å and *U*_iso_(H) = 0.05 Å^2^. C-bound H atoms were positioned geometrically and treated as riding on their parent atoms with C-H = 0.96 Å and *U*_iso_(H) = 1.5*U*_eq_(C).

### Crystal data

**Table:**

[Na(C_3_H_3_N_2_S_3_)(H_2_O)_2_]	*F*(000) = 228
*M**_r_* = 222.28	*D*_x_ = 1.644 Mg m^−^^3^
Monoclinic, *P*2_1_/*m*	Mo *K*α radiation, λ = 0.71073 Å
Hall symbol: -P 2yb	Cell parameters from 1437 reflections
*a* = 7.5794 (8) Å	θ = 2.4–27.9°
*b* = 6.9736 (6) Å	µ = 0.83 mm^−^^1^
*c* = 8.6879 (12) Å	*T* = 298 K
β = 102.027 (1)°	Block, colourless
*V* = 449.13 (9) Å^3^	0.39 × 0.27 × 0.15 mm
*Z* = 2	

### Data collection

**Table:**

Siemens SMART CCD area-detector diffractometer	862 independent reflections
Radiation source: fine-focus sealed tube	728 reflections with *I* > 2σ(*I*)
graphite	*R*_int_ = 0.017
φ and ω scans	θ_max_ = 25.0°, θ_min_ = 2.4°
Absorption correction: multi-scan (*SADABS*; Sheldrick, 1996)	*h* = −7→9
*T*_min_ = 0.739, *T*_max_ = 0.886	*k* = −8→8
2256 measured reflections	*l* = −10→10

### Refinement

**Table:**

Refinement on *F*^2^	Primary atom site location: structure-invariant direct methods
Least-squares matrix: full	Secondary atom site location: difference Fourier map
*R*[*F*^2^ > 2σ(*F*^2^)] = 0.024	Hydrogen site location: inferred from neighbouring sites
*wR*(*F*^2^) = 0.064	H-atom parameters constrained
*S* = 1.07	*w* = 1/[σ^2^(*F*_o_^2^) + (0.027*P*)^2^ + 0.2249*P*] where *P* = (*F*_o_^2^ + 2*F*_c_^2^)/3
862 reflections	(Δ/σ)_max_ = 0.001
64 parameters	Δρ_max_ = 0.28 e Å^−^^3^
0 restraints	Δρ_min_ = −0.21 e Å^−^^3^

### Special details

**Table:**

Geometry. All e.s.d.'s (except the e.s.d. in the dihedral angle between two l.s. planes) are estimated using the full covariance matrix. The cell e.s.d.'s are taken into account individually in the estimation of e.s.d.'s in distances, angles and torsion angles; correlations between e.s.d.'s in cell parameters are only used when they are defined by crystal symmetry. An approximate (isotropic) treatment of cell e.s.d.'s is used for estimating e.s.d.'s involving l.s. planes.
Refinement. Refinement of *F*^2^ against ALL reflections. The weighted *R*-factor *wR* and goodness of fit *S* are based on *F*^2^, conventional *R*-factors *R* are based on *F*, with *F* set to zero for negative *F*^2^. The threshold expression of *F*^2^ > σ(*F*^2^) is used only for calculating *R*-factors(gt) *etc*. and is not relevant to the choice of reflections for refinement. *R*-factors based on *F*^2^ are statistically about twice as large as those based on *F*, and *R*- factors based on ALL data will be even larger.

### Fractional atomic coordinates and isotropic or equivalent isotropic displacement parameters (Å^2^)

**Table:**

	*x*	*y*	*z*	*U*_iso_*/*U*_eq_	Occ. (<1)
Na1	0.07064 (15)	0.2500	0.93636 (13)	0.0400 (3)	
N1	0.1651 (3)	0.2500	0.4084 (3)	0.0339 (5)	
N2	0.1364 (3)	0.2500	0.6700 (2)	0.0291 (5)	
O1	0.16585 (17)	−0.0019 (2)	1.13259 (15)	0.0413 (4)	
H1A	0.1666	0.0502	1.2212	0.050*	
H1B	0.2701	−0.0493	1.1374	0.050*	
S1	0.37894 (9)	0.2500	0.51077 (9)	0.0386 (2)	
S2	−0.17326 (9)	0.2500	0.46612 (9)	0.0378 (2)	
S3	0.46031 (10)	0.2500	0.86933 (9)	0.0448 (2)	
C1	0.0626 (3)	0.2500	0.5131 (3)	0.0283 (6)	
C2	0.3165 (3)	0.2500	0.6910 (3)	0.0310 (6)	
C3	−0.2220 (4)	0.2500	0.2548 (3)	0.0470 (8)	
H3A	−0.3503	0.2500	0.2163	0.071*	
H3B	−0.1709	0.1376	0.2175	0.071*	0.50
H3C	−0.1709	0.3624	0.2175	0.071*	0.50

### Atomic displacement parameters (Å^2^)

**Table:**

	*U*^11^	*U*^22^	*U*^33^	*U*^12^	*U*^13^	*U*^23^
Na1	0.0488 (7)	0.0382 (7)	0.0350 (6)	0.000	0.0134 (5)	0.000
N1	0.0315 (12)	0.0391 (14)	0.0321 (13)	0.000	0.0085 (10)	0.000
N2	0.0261 (11)	0.0318 (12)	0.0302 (12)	0.000	0.0074 (9)	0.000
O1	0.0393 (8)	0.0461 (9)	0.0374 (8)	0.0029 (6)	0.0054 (6)	−0.0041 (7)
S1	0.0274 (4)	0.0514 (5)	0.0394 (4)	0.000	0.0122 (3)	0.000
S2	0.0256 (4)	0.0444 (5)	0.0423 (4)	0.000	0.0043 (3)	0.000
S3	0.0319 (4)	0.0626 (6)	0.0364 (4)	0.000	−0.0009 (3)	0.000
C1	0.0286 (13)	0.0214 (13)	0.0350 (15)	0.000	0.0067 (11)	0.000
C2	0.0280 (13)	0.0295 (15)	0.0358 (15)	0.000	0.0070 (11)	0.000
C3	0.0439 (17)	0.0468 (19)	0.0426 (18)	0.000	−0.0088 (14)	0.000

### Geometric parameters (Å, °)

**Table:**

Na1—O1^i^	2.4493 (16)	N2—C1	1.361 (3)
Na1—O1	2.4493 (16)	O1—Na1^ii^	2.4736 (16)
Na1—N2	2.467 (2)	O1—H1A	0.85
Na1—O1^ii^	2.4736 (16)	O1—H1B	0.85
Na1—O1^iii^	2.4736 (16)	S1—C2	1.727 (3)
Na1—S3	3.1271 (14)	S2—C1	1.749 (3)
Na1—Na1^ii^	3.8756 (10)	S2—C3	1.796 (3)
Na1—Na1^iv^	3.8756 (10)	S3—C2	1.698 (3)
N1—C1	1.314 (3)	C3—H3A	0.96
N1—S1	1.679 (2)	C3—H3B	0.96
N2—C2	1.340 (3)	C3—H3C	0.96
			
O1^i^—Na1—O1	91.62 (8)	Na1^ii^—Na1—Na1^iv^	128.23 (6)
O1^i^—Na1—N2	124.42 (5)	C1—N1—S1	106.14 (18)
O1—Na1—N2	124.42 (5)	C2—N2—C1	109.3 (2)
O1^i^—Na1—O1^ii^	139.79 (5)	C2—N2—Na1	105.76 (16)
O1—Na1—O1^ii^	76.14 (5)	C1—N2—Na1	144.92 (16)
N2—Na1—O1^ii^	92.90 (6)	Na1—O1—Na1^ii^	103.86 (5)
O1^i^—Na1—O1^iii^	76.14 (5)	Na1—O1—H1A	105.7
O1—Na1—O1^iii^	139.79 (5)	Na1^ii^—O1—H1A	112.7
N2—Na1—O1^iii^	92.90 (6)	Na1—O1—H1B	116.7
O1^ii^—Na1—O1^iii^	88.79 (7)	Na1^ii^—O1—H1B	111.0
O1^i^—Na1—S3	88.68 (4)	H1A—O1—H1B	106.9
O1—Na1—S3	88.68 (4)	N1—S1—C2	93.66 (12)
N2—Na1—S3	56.09 (5)	C1—S2—C3	102.79 (14)
O1^ii^—Na1—S3	128.30 (4)	C2—S3—Na1	73.65 (9)
O1^iii^—Na1—S3	128.30 (4)	N1—C1—N2	121.0 (2)
O1^i^—Na1—Na1^ii^	120.36 (6)	N1—C1—S2	124.2 (2)
O1—Na1—Na1^ii^	38.29 (3)	N2—C1—S2	114.86 (18)
N2—Na1—Na1^ii^	112.92 (3)	N2—C2—S3	124.5 (2)
O1^ii^—Na1—Na1^ii^	37.85 (3)	N2—C2—S1	109.90 (19)
O1^iii^—Na1—Na1^ii^	117.98 (6)	S3—C2—S1	125.60 (16)
S3—Na1—Na1^ii^	112.39 (3)	S2—C3—H3A	109.5
O1^i^—Na1—Na1^iv^	38.29 (3)	S2—C3—H3B	109.5
O1—Na1—Na1^iv^	120.36 (6)	H3A—C3—H3B	109.5
N2—Na1—Na1^iv^	112.92 (3)	S2—C3—H3C	109.5
O1^ii^—Na1—Na1^iv^	117.98 (6)	H3A—C3—H3C	109.5
O1^iii^—Na1—Na1^iv^	37.85 (4)	H3B—C3—H3C	109.5
S3—Na1—Na1^iv^	112.39 (3)		

Symmetry codes: (i) *x*, −*y*+1/2, *z*; (ii) −*x*, −*y*, −*z*+2; (iii) −*x*, *y*+1/2, −*z*+2; (iv) −*x*, −*y*+1, −*z*+2.
